# Urban bumblebees diversify their foraging strategy to maintain nutrient intake

**DOI:** 10.1098/rspb.2025.0639

**Published:** 2025-05-21

**Authors:** Simonetta Selva, Marco Moretti, Fabian A. Ruedenauer, Alexander Keller, Bertrand Fournier, Sara Diana Leonhardt, Helen Eggenberger, Joan Casanelles Abella

**Affiliations:** ^1^ Swiss Federal Institute for Forest Snow and Landscape Research, Birmensdorf, Switzerland; ^2^ ETH Zurich, Zurich, Zürich, Switzerland; ^3^ Community Ecology, Swiss Federal Research Institute WSL, Bellinzona, Switzerland; ^4^ Plant-Insect-Interactions, Technische Universität München, Freising, Germany; ^5^ Cellular and Organismic Networks, LMU, Planegg-Martinsried, Germany; ^6^ University of Potsdam, Potsdam, Brandenburg, Germany; ^7^ Urban Productive Ecosystems, TUM, Munich, Germany

**Keywords:** feeding behaviour, intraspecific trait variability, land-use changes, plant–pollinator interactions, pollination, urban biodiversity

## Abstract

Anthropogenic ecosystems can alter individual functions and ecological processes such as resource use and species interactions. While variability of morphological traits involved in diet and resource use has been observed between urban and non-urban populations of pollinators, the consequences on the dietary and pollen-transport patterns remain poorly understood. Here, we investigate the variability in the diet breadth of rural and urban individuals of two bumblebee species and the consequences for nutrient intake and pollen transport. We show that urban bumblebees exhibit a larger diet breadth than their rural counterparts, driven by the enhanced floral diversity in cities. However, we found that the nutrient intake remained similar across urban and rural ecosystems, indicating that bumblebees' foraging strategies can be adapted in terms of diet breadth to maintain intake and ratios of critical nutrients. We also found distinct pollen-transport patterns between urban and rural individuals, with urban individuals being more dissimilar than rural ones in the transported pollen both in the body and in the leg baskets. Our findings highlight the importance of considering complementary facets of species’ diet and interactions when assessing the effects of anthropogenic ecosystems.

## 1. Introduction

Urban and rural ecosystems have altered the foraging landscape for animals, impacting dietary patterns, such as diet breadth and nutrient intake, and shaping foraging strategies and traits. Bumblebees, as central-place foragers, are ideal for studying these shifts since resources change significantly between urban and rural areas [1]. Bumblebee foraging traits, like tongue length and body size, are linked to feeding specialization, which can vary within species as floral resource compositions shift [2]. These trait differences influence plant visitation patterns and interaction networks, potentially affecting diet, nutrient intake and pollination services.

Urban and rural areas have distinct floral diversity patterns including the composition and structure of species and nutrients [3], and can have different effects on the dietary patterns and pollen-transport of urban and rural bumblebees. According to the optimal foraging theory [4], bumblebee individuals are expected to have foraging strategies and behaviours that maximize their colonies’ net yield of energy [5]. Thus, the foraging distance will depend on the degree to which the resources are accessible (or isolated) and how evenly they are distributed [6]. Such predictions have been confirmed in bumblebees [7] with flight duration and flight distance negatively related with the coverage of green areas around colonies in several species [8].

How different foraging strategies affect nutrient intake in bumblebees remains unknown. In rural areas, bumblebees have been found to have a consistent diet breadth [9]. Nevertheless, in urban areas, bumblebees may have larger diet breadths, resulting from the visitation of a larger and probably more distinct plant diversity [10] than their rural counterparts. This is supported by the diet diversification hypothesis [11,12]. However, a broader diet does not always ensure better nutrient intake [1,13], as the nutritional value and toxicity of pollen varies widely between plant species [14]. Bumblebees often select pollen with specific nutrient profiles, focusing on low fatty acid (FA) and high amino acid (AA) content, and aim to balance protein-to-lipid ratios [15,16]. These dietary adaptations help bumblebees meet strict nutritional needs to optimize their fitness, though such requirements may vary across species and might be altered in anthropogenically modified ecosystems. In that regard, if diet breadth is expanded in cities there are three possible outcomes regarding nutrient intake: (i) nutrient intake remains similar, (ii) nutrient intake increases in cities together with diet breadth and (iii) nutrient intake deteriorates in cities.

Moreover, the altered structure, composition, distribution and accessibility of the floral resources might further shape bumblebee foraging decisions [6]. When preferred floral resources are scarcer, bumblebees might select suboptimal floral resources that require less energy investment to find and access, or that are less preferred by other pollinating individuals to avoid competition [17]. When this results in shortages of certain nutrients, bees may be forced to increase the number of plant species they visit for compensation, resulting in more plant species visited per individual [18,19].

Novel insights in bumblebee foraging strategies can be obtained by complementing the study of the diet breadth and nutrient intake with ecological networks [20]. Bumblebee–plant interaction networks can be assembled for the two pollen-transport structures (leg baskets and body) to further understand how urban and rural landscapes are shaping bumblebee foraging strategies. Particularly, metrics quantifying the number of resources collected (e.g. generality) or the degree to which individuals collect similar resources (e.g. niche overlap) could be used to further evaluate how optimal are foraging landscapes. For example, in suboptimal landscapes, increasing the number of plants visited could lead to a lower degree of niche overlap in the collected floral resources [18,19].

Here, we compare the foraging strategies of two common intermediate generalist bumblebee species [2], *Bombus lapidarius* (Linnaeus, 1761) and *Bombus pascuorum* (Scopoli, 1763), in two distinct anthropogenically modified ecosystems, namely urban and rural areas. Specifically, we have four main goals. First, compare the diet breadth between urban and rural bumblebee populations. Second, test the influence of floral resource richness in the study sites and the variability in morphological traits (i.e. mouthparts and body lengths) on diet breadth between urban and rural populations. Third, compare the nutrient intake patterns of two key macronutrient groups between urban and rural populations. Fourth, compare the pollen-transport patterns (*sensu* [21], assessed as the plant-individual bumblebee interaction networks) in urban and rural populations.

We expect that our studied species have certain requirements regarding FAs and AAs [15,16,22], and certain conservatism for specific plant families as seen in natural and semi-natural ecosystems [2]. Simultaneously, according to the optimal foraging theory, we expect bumblebees to adopt foraging strategies that maximize the energy return while reducing the associated costs, determined by the floral resource richness and the variability in morphological traits. Thus, we expect urban bumblebees to visit a larger number of plant species and to show a lower niche overlap and a higher generality [23]. Regarding pollen transport [24,25], we expect urban bumblebees to have a larger number of plants visited in urban bumblebees, lower niche overlap and higher generality (electronic supplementary material, figure S1). Based on this, we consider three scenarios of nutritional intake (electronic supplementary material, figure S1):

1. *Nutrient intake maintenance:* nutrient intake is similar in urban and rural areas, indicating strong overarching preferences for certain nutrients as well as ratios between nutrient types (e.g. proteins and lipids) [15,16].
2. *Urban advantage due to diversification:* nutrient intake is different between urban and rural areas, indicating a distinct foraging, resulting in improved nutrition [26].
3. *Urban disadvantage:* alternatively to scenario (2), the distinct nutrient intake between urban and rural areas might deteriorate nutrition in cities.

## 2. Material and methods

### (a) Study sites and bumblebee sampling

We sampled bumblebees in urban and rural areas in three Swiss regions (hereinafter regions), specifically in the Cantons of Basel, Bern and Zurich (electronic supplementary material, text S1, figure S2 and table S1). For each region, we selected three sampling sites in both urban and rural areas (except for rural Bern where only one sampling site could be selected), with a total of 16 site. Within each region, each sampling site was defined by a circle of 800 m radius that represented the foraging ranges of the studied species. We studied the common carder bee *B. pascuorum* and the red-tailed bumblebee *B. lapidarius*. These two bumblebees are common in the Swiss lowlands and present in both urban and rural areas. They are both generalists, although *B. pascuorum* has a longer tongue (longer tongues are more link to specialization) than *B. lapidarius*.

Bumblebees were collected by hand-netting following targeted sampling, in the highest activity months of the season for both species, that is, July to mid-August, in 2016. Within each 800 m radius, we collected 30−40 individuals per species, except for one urban site in Bern where only three individuals of *B. lapidarius* were found. Sampling efforts were standardized across all sites and conducted during peak bumblebee activity hours (09.00−17.00) and under optimal weather conditions. We walked the entire 800 m radius searching for the targeted bumblebee species until we had obtained 30−40 individuals. Only active foragers were collected. Species identity of all collected individuals was verified in the laboratory, and specimens that could not be clearly identified were removed. Additional details are presented in Eggenberger *et al*. [23], Casanelles-Abella *et al*. [27] and electronic supplementary material, text S1.

### (b) Pollen collection and metabarcoding

We extracted pollen from the corbicula and the body of the bumblebees separately (see electronic supplementary material, Text S2). In total we used 152 individuals of *B. pascuorum* and 238 individuals of *B. lapidarius* across all sampling sites from which we found pollen in both the corbicula and the body (electronic supplementary material, tables S2 and S3). This resulted in 390 samples of corbicula pollen and 390 samples of body pollen in total.

DNA metabarcoding (isolation, amplification, and sequencing) of pollen samples was performed by AllGenetics laboratories (AllGenetics & Biology SL; A Coruña, Spain). In summary, the ITS2 region was amplified according to existing protocols [28,29]. The libraries were then purified, pooled, and sequenced on an Illumina NovaSeq platform. Taxonomy was assigned to amplicon sequence variants (ASVs) using a pre-trained classifier, and filtering steps were applied to remove singletons and correct for mistagging issues. Finally, we calculated the relative abundance per plant species, that is, the frequency of an ASV within each sample. Regarding bioinformatics, we followed the pipeline described at https://github.com/chiras/metabarcoding_pipeline [30]. Additional details are described in Casanelles-Abella *et al*. [27] and in electronic supplementary material, text S3.

### (c) Nutritional analyses

We focused on two critical macronutrients for bumblebee health and fitness: AAs and FAs [31]. To have a sufficient pollen mass to perform the nutritional analyses, we pooled pollen (from the leg baskets) from different bumblebee individuals within study sites and for each species separately (electronic supplementary material, table S3).

We used ion exchange chromatography (IEC: Biochrom 20 Plus Amino Acid Analyzer) to analyse protein-bound AAs in pollen, following the protocol outlined by Kriesell *et al*. [32], and explained in electronic supplementary material, text S4. The total protein content was calculated as the sum of all AAs. Therefore, in this study, the AA content always refers to the total content of protein-bound AAs. Moreover, we also calculated the total content of essential AAs, which cannot be synthesized by animals and have to be obtained exclusively from the diet, and non-essential AAs, which can be synthesized by animals (electronic supplementary material, table S4). Additionally, we also calculated the ratio between essential and non-essential AAs (electronic supplementary material, table S4).

The analysis of FAs followed the protocol outlined by Villagómez *et al*. [33] and explained in electronic supplementary material, text S4. We also calculated the content and ratios of specific types of FAs relevant for bee nutrition, survival and health [34–36], particularly, the content of total, saturated, non-saturated, omega 3, omega 6 and omega 9 FAs, and the ratios between saturated versus unsaturated FA and between omega 3 versus omega 6 (electronic supplementary material, table S4). These different metrics on FAs can be related to nutrition and health.

### (d) Floral resource richness in the landscape

Floral resource richness in the landscape was inferred using plant species richness, as abundance and biomass were not available. Particularly, floral resource richness was assessed by compiling a list of plants occurring at each site within a 1500 m buffer following bumblebee foraging ranges using data from two sources: the Global Biodiversity Information Facility (GBIF) [37] and the National Data and Information Centre on the Swiss Flora (Infoflora) [38]. Infoflora, which monitors plant diversity across Switzerland, provided data on 1759 mostly native and invasive species. To account for non-native, non-invasive ornamental species often found in cities, we added observations from GBIF [37] with 443 additional species.

### (e) Traits

We used plant floral traits to understand the mechanisms behind the flower choice of bumblebees [39]. We collected data on four functional traits, including flowering duration, growth form, blossom class (a classification of the flowers/inflorescences based on the accessibility of the rewards [40]) and nectar sugar concentration [41] and one descriptive trait, namely, the origin status (electronic supplementary material, table S5). Plant traits were collected from multiple published, open-source datasets [40–42]. As the availability of sugar concentration data was limited, we retrieved sugar concentration data for the most abundant plant species among others (electronic supplementary material, figure S3), which together represented 79% of the relative abundance of the plant species visited by bumblebees. We selected three non-correlated (<0.7; electronic supplementary material, figure S4) out of the original four functional traits for computing the functional diversity indices. The final selected traits were flowering duration, blossom class and sugar concentration of the nectar.

We used the bumblebee morphological traits measured by Eggenberger *et al*. [23], which are directly or indirectly linked to foraging behaviour (electronic supplementary material, table S6). Specifically, we used the intertegular distance, proboscis length, forewing length and corbicula length. Due to the existing allometric relationships between body parts, for forewing, proboscis and corbicula length, we calculated the ratio between the traits and intertegular distance (i.e. proboscis ratio, corbicula ratio, forewing length ratio).

### (f) Statistical analysis

We conducted all analyses in R version 4.2.1 [43].

#### (i) Diversity metrics for diet breadth

Diet breadth, defined as the total number of resources in the diet [44], has been assessed in bees using taxonomic richness [45]. To allow for complementary interpretation, we used taxonomic, functional and phylogenetic metrics to infer bumblebee diet breadth. We used plant species richness for plant taxonomic diversity [45]. Regarding functional diversity, we calculated functional richness, functional evenness, and functional divergence indices, using the package ‘FD’ version 1.0−12.1 by Laliberté & Legendre [46]. Finally, we calculated multidimensional phylogenetic metrics, specifically, phylogenetic variability, phylogenetic richness, phylogenetic evenness, and phylogenetic clustering, using the package ‘picante’ version 1.8.2 by Kembel *et al.* [47]*.* We used the phylogeny in Jin & Qian [48]. For functional metrics, bumblebee individuals with less than four plant species in the collected pollen were excluded as the convex hull could not be computed (*n* = 154 individuals; electronic supplementary material, table S3). For phylogenetic metrics, bumblebee individuals with less than three species were removed, leading to a total number (*n* = 264 individuals; electronic supplementary material, table S3).

#### (ii) Comparing diet breadth and nutrient intake in urban and rural areas

For diet breadth, we used the computed taxonomic, functional, and phylogenetic metrics at the individual level. For nutrient intake, we considered the different content and ratios of AAs and FAs (see §2c). We used linear mixed-effects models with landscape (urban and rural) as a fixed factor and region as a random effect. Models were done separately for the two bumblebee species. To correct for multiple comparisons, we used the Benjamini–Hochberg correction.

#### (iii) Influence of landscape type, floral resources and bee morphological traits on diet breadth and nutrient intake

We modelled the direct and indirect effects that shape the collected pollen in the corbicula. Specifically, as predictors we used the landscape type (i.e. urban and rural), the floral resources at the landscape scale within 1500 m (plant species richness) and two uncorrelated bee morphological traits related to foraging, that is, intertegular distance and proboscis ratio (electronic supplementary material, figure S5). We used multilevel structural equation modelling, implemented in the *piecewiseSEM* package version 2.3 [49], following Shipley [50] and Casanelles-Abella *et al.* [51]. All variables except the species richness in the pollen were centred and scaled before the analyses. We used generalized linear mixed effects models (GLMMs) as composite SEMs. Particularly, we modelled the species richness in the pollen with a Poisson distribution and the plant richness at the landscape scale, the intertegular distance and the proboscis ratio with a Gaussian distribution. Missing paths in the SEM were checked with Shipley’s d-separation test [52] and independence claims (the basis set) summarized with the Fisher’s C statistic (*p*  <  0.05 [50]).

The final SEM model included four components. The main model (component 1) for plant species richness in the collected pollen included the available resources at the landscape scale (within 1500 m radius), the intertegular distance and the proboscis ratio as predictors. In addition, we also assessed the influence of the landscape type on the available floral resources at the landscape scale (component 2), that is, plant species richness at the landscape scale. We also assessed the influence of landscape type (urban and rural) and available resources at the landscape scale on the intertegular distance (component 3) and the proboscis ratio (component 4). Initially, we used a nested random factor with site nested to region. However, the site variance was nearly 0 and the models fail to converge. Thus, in all models, we used region as random factor. Finally, we checked model assumptions, as well as potential spatial autocorrelation patterns in the response variables and the model residuals. Additionally, we conducted GLMMs on uncorrelated functional and phylogenetic diversity metrics (i.e. functional evenness, functional dispersion, phylogenetic variance) using similar SEMs.

#### (iv) Pollen transport patterns

We studied pollen-transport patterns classifying the collected plants according to the pollen-transport structure they were found in, that is, in the leg (i.e. corbicula), in the body or in both structures. We calculated the proportion of plants in these three categories separately per bumblebee species and landscape type. Additionally, to further explore both pollen-transport and dietary patterns, we built bipartite pollen-transport networks and calculated different network metrics using the packages bipartite [53] and igraph [54], considering as nodes the bumblebee individuals and the plant species respectively. We assembled the networks separately for each species, landscape type and two pollen-transport structures (i.e. body and leg baskets). We assembled networks using the relative abundances as a measure of strength of interaction. For each network, we calculated the mean number of links per bumblebee individual and per plant species, the niche overlap between bumblebee individuals (a metric of the degree to which the different bumblebee individuals use similar resources) and the generality (a measure of how many different species an individual bumblebee interacts with within the network). Both niche overlap and generality can be used to evaluate how competitive or optimal the foraging landscapes are [55].

## 3. Results

We found a total of 231 plant species belonging to 47 families across all study sites visited by the two bumblebee species. Although having a longer tongue, *B. pascuorum* foraged on more plant species than *B. lapidarius* (*B. pascuorum* = 176 species, *B. lapidarius* = 157 species). The two bumblebee species predominantly foraged on plants from the family Fabaceae (electronic supplementary material, figures S6 and S7, *B*. *pascuorum* = 84 %, *B. lapidarius* = 81%), with the species *Trifolium pratense* (*B. pascuroum* = 67%, *B. lapidarius* = 32%) and *T. repens* (*B. pascuroum* = 9%, *B. lapidarius* = 22%) and *Lotus corniculatus* (*B. pascuroum* = 4%, *B. lapidarius* = 23%) representing a substantial part of the collected pollen.

### (a) Differences in the diet breadth

**Figure 1.**
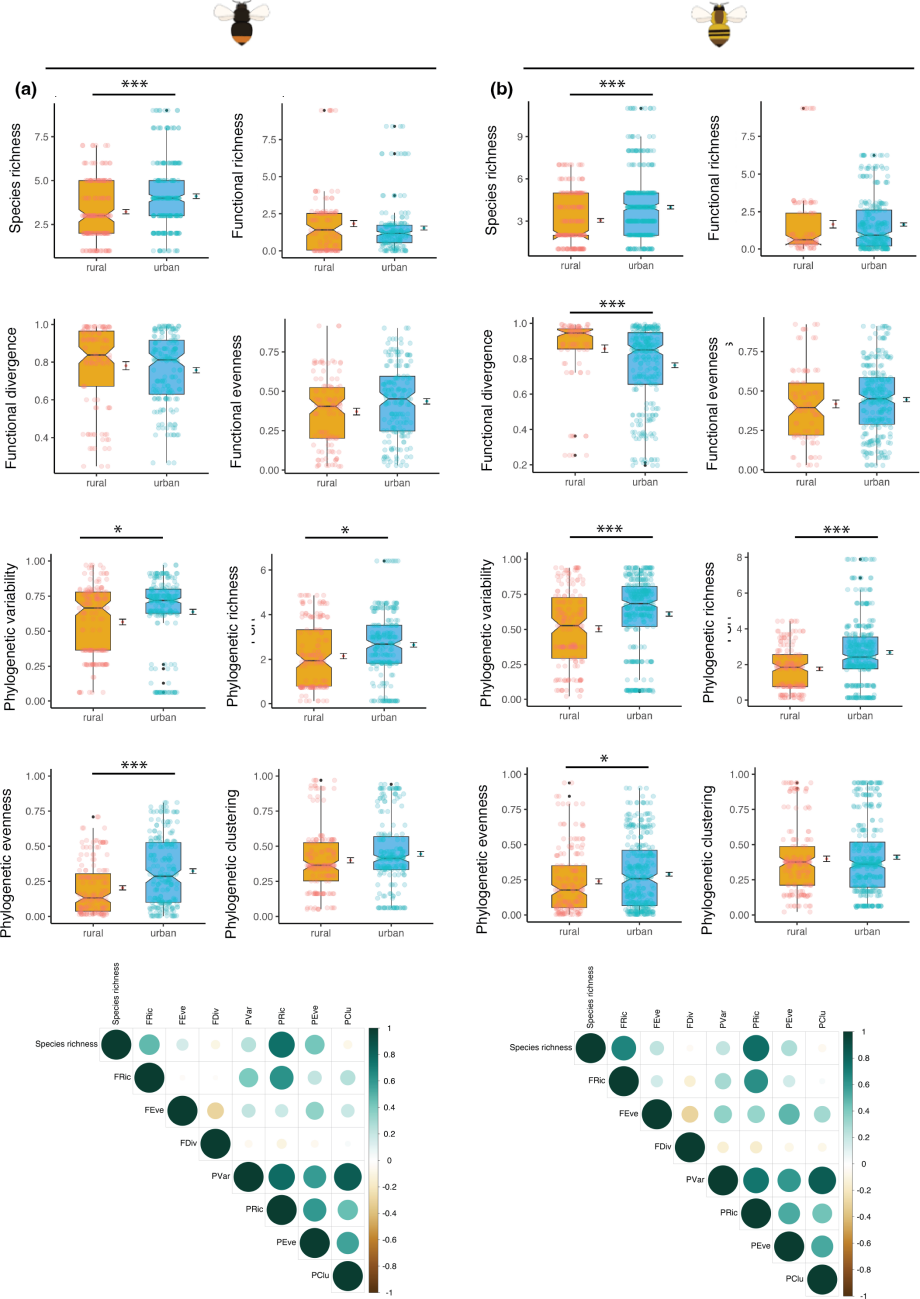
Differences in the dietary patterns in urban and rural bumblebee populations. Boxplots depicting the differences between the pollen taxonomic (species richness), functional (functional richness, FRich; functional evenness, FEve; functional dispersion, FDis), and phylogenetic (phylogenetic richness, Pric, phylogenetic variability, Pvar, phylogenetic evenness, Peve, phylogenetic clustering, Pclu) diversity metrics between urban and rural populations of *B. lapidarius* (a, left panels) and *B. pascuorum* (b, right panels). Notches indicate the 95% confidence interval of the median. Additionally, on the right side of each boxplot, the mean ± the standard error is also presented. Differences between the means were tested using generalized linear lixed effects models with Benjamini–Hochberg correction for multiple testing. Correlation plots show the correlations between the taxonomic, functional and phylogenetic metrics. Significance levels: *: 0.05>*p-*value>0.01, **: 0.01>*p-*value>0.001, ***: *p*‐value<0.001.

Urban bumblebees had a wider diet breadth than their rural counterparts (figure 1; electronic supplementary material, figures S6–S8). First, urban bumblebees collected a larger number of plant species in their pollen load than their rural counterparts (*B. pascuroum:* urban *=* 69*,* rural = 34; *B. lapidarius:* urban = 55, rural = 21; figure 1; electronic supplementary material, table S7). Urban bumblebees also visited a wider range of plant families, while rural bumblebees tended to forage from a more limited number of families (*B. pascuroum:* urban *=* 22*,* rural = 11; *B. lapidarius:* urban = 20, rural = 9; electronic supplementary material, tables S7 and S8, figures S6 and S7). Second, we found that urban bumblebees visited a greater diversity of structural blossom classes (electronic supplementary material, figure S8) and a slightly higher percentage of woody plants (electronic supplementary material, figure S8) and non-native species (electronic supplementary material, figure S8), particularly in Zurich. The larger diet breadth of urban bumblebees as compared to rural ones was visible in most phylogenetic metrics of both bumblebee species (electronic supplementary material, table S8; figure 1), and in the functional divergence in *B. pascuorum* (electronic supplementary material, table S8; figure 1). This indicates that rural bumblebees foraged on a reduced number of plants that in addition were phylogenetically closely related.

### (b) Influence of landscape type, floral resource richness and bee morphological traits on diet breadth

**Figure 2.**
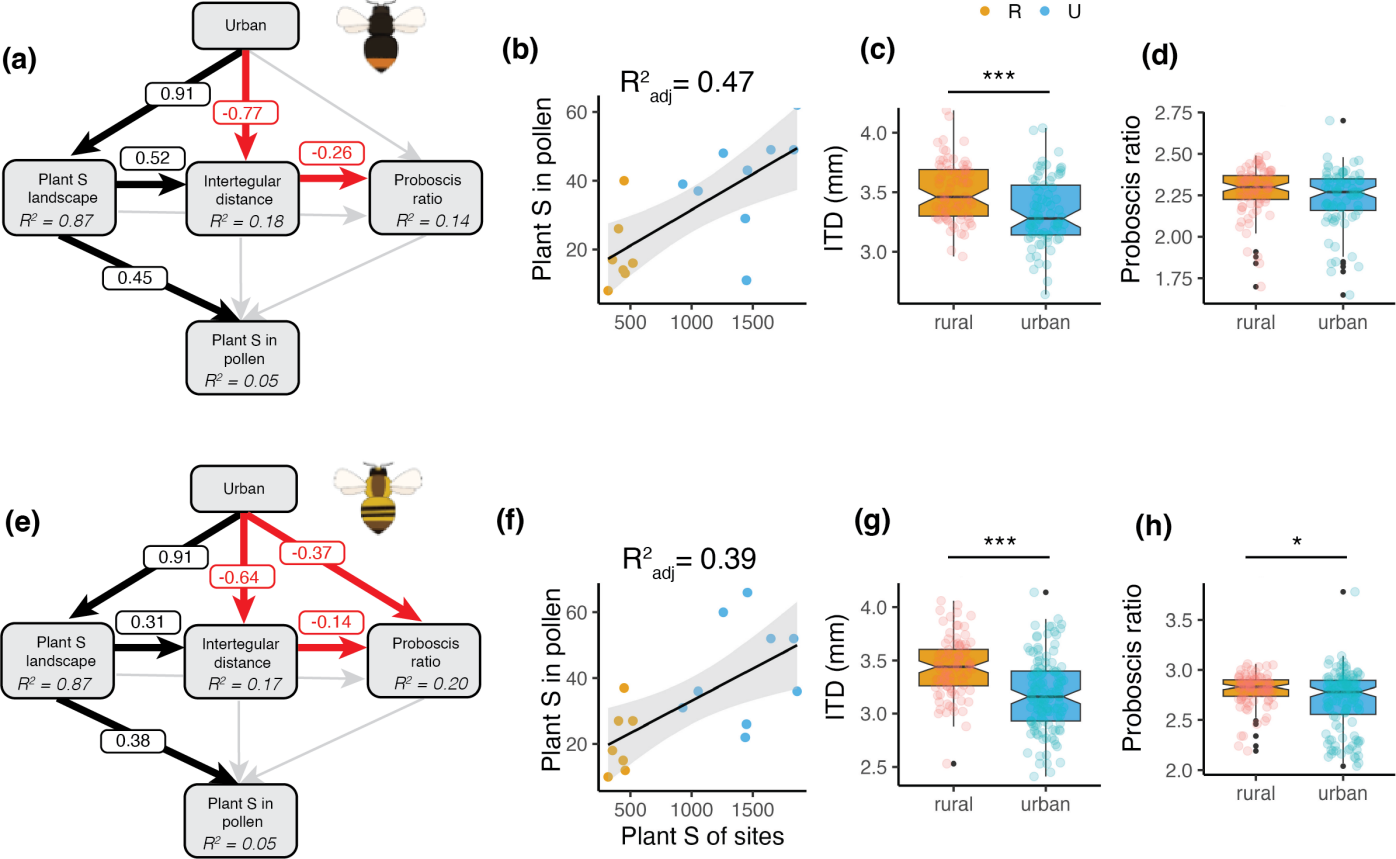
Drivers of bumblebee dietary patterns. (a,e) Piecewise structural equation modelling (pSEM) depicting the direct and indirect effects of landscape type (i.e. urban and rural), floral resources within a 1500 m buffer (i.e. Plant S of sites), and morphological traits related to foraging (i.e. Intertegular distance ITD, and proboscis ratio that results from dividing the proboscis length and the intertegular distance) on the plant species richness of the pollen (Plant S in pollen) collected in the corbicula of individuals of *B. lapidarius* (a) and *B. pascuorum* (e). The pSEM also includes three models explaining the influence of the landscape type on the floral resources in the landscape and the influence of the landscape type and the floral resources within a 1500 m buffer on the intertegular distance and the proboscis ratio. Numbers show standardized path coefficients for significant pathways. Positive paths are depicted in black, negative in red, and nonsignificant (*p*  >  0.05) in grey. For each response variable, the *R*^2^ is provided inside the box. *B. lapidarius*: Fisher’s C = 0.879, *p*‐value = 0.644. *B. pascuorum:* Fisher’s C = 0.628, *p*‐value = 0.731. Additional pSEMs with the functional and phylogenetic diversity metrics are shown in electronic supplementary material, table S8. (b,f) Linear models depicting the relationship between total species richness in the pollen collected and plant species richness in the landscape at different radii. Shaded bands depict the 95% CI. Points represent the study sites. Species richness in the pollen is calculated by pooling all the bumblebee individuals per study site. (c,d,g,h) Boxplots depicting the differences in the intertegular distance (c,g) and proboscis ratio (d,h) between rural and urban bumblebee individuals. Notches indicate the 95% CI of the median. S = species richness. For pSEM, R^2^ is the conditional R^2^.

Our results revealed a main role of floral resource richness at the landscape scale (inferred as the plant species richness per site) in shaping diet breadth of both bumblebee species (figure 2). In both cases, floral resource richness at the landscape scale positively increased the species richness (*B. lapidarius*: 0.376 ± 0.115, *p*‐value = 0.001; *B. pascuorum*: 0.449 ± 0.139, *p*‐value = 0.001; electronic supplementary material, table S9). Furthermore, floral resource richness at the landscape scale was much larger in urban areas (mean, min–max: 1457, 947−1884 plant species) than in rural areas (433, 322–530 plant species) (figure 2; electronic supplementary material, figure S9 and table S9). Conversely, plant diet breadth patterns were not affected by bumblebee morphological traits, that is, intertegular distance and proboscis ratio (figure 2). Furthermore, intertegular distance was lower in urban landscapes and was positively correlated with plant species richness at the landscape scale in both species (figure 2; electronic supplementary material, figure S10 and table S9). Proboscis ratio in *B. pascuorum* was also lower in urban landscapes. Finally, the GLMMs on the plant functional and phylogenetic metrics did not indicate any significant effect of plant resources and bee morphological traits in shaping the diversity metrics (electronic supplementary material, figure S10, table S10).

### (c) Differences in the nutrient intake

**Figure 3.**
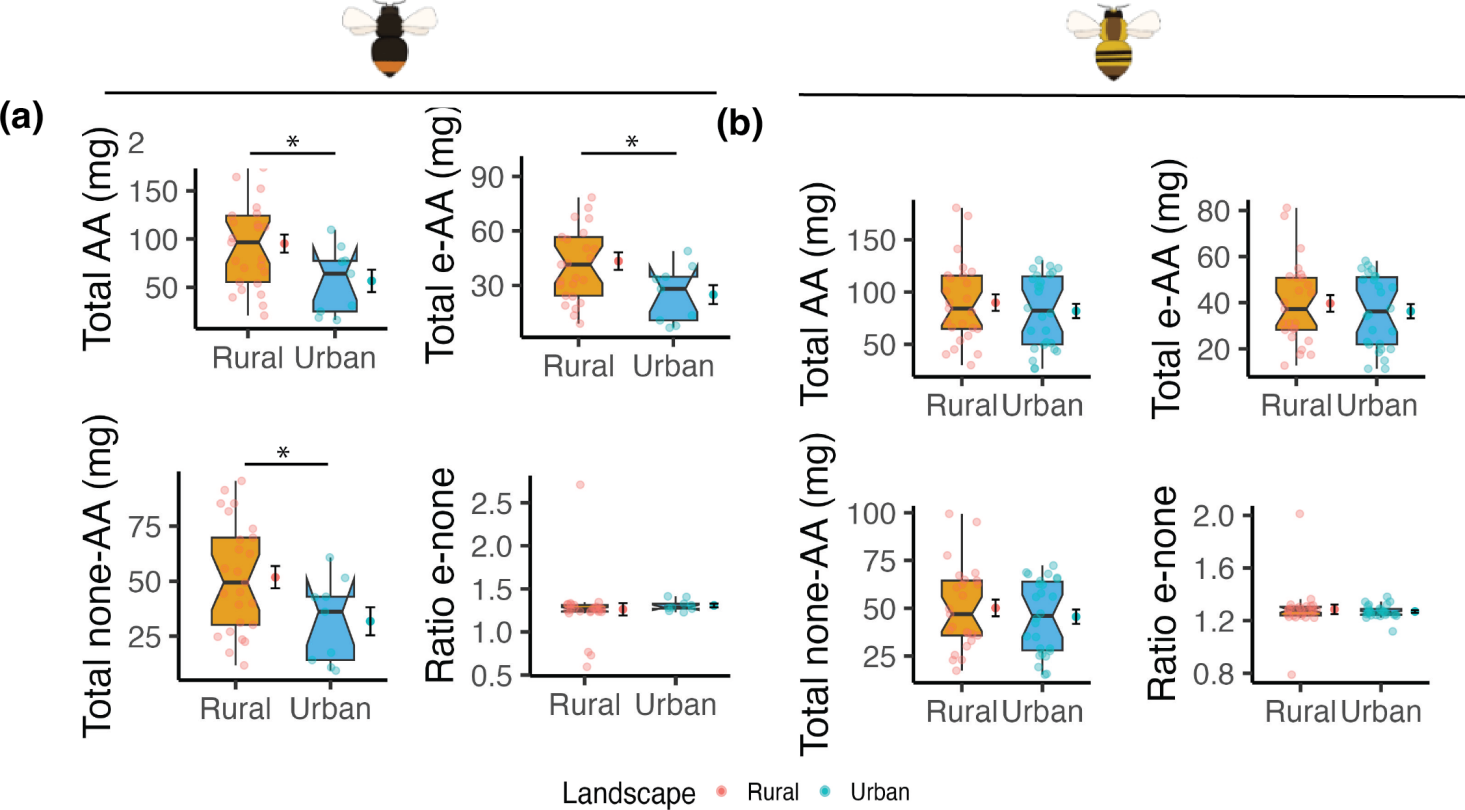
Urban and rural bumblebees have a similar amino acid (AA) intake. Boxplots of the AA composition in the pollen from the leg baskets of urban and rural bumblebees of (a) *Bombus lapidarius* and (b) *Bombus pascuorum*. For simplicity, AAs have been grouped in four main groups. Notches indicate the 95% confidence interval of the median. Additionally, on the right side of each boxplot, the mean ± the standard error is also presented. Differences between the means were tested using generalized linear mixed effects models with Benjamini–Hochberg correction for multiple testing. e = essential; none = non-essential. Significance level is set at *p* < 0.05. *: 0.05 > *p* > 0.001.

**Figure 4.**
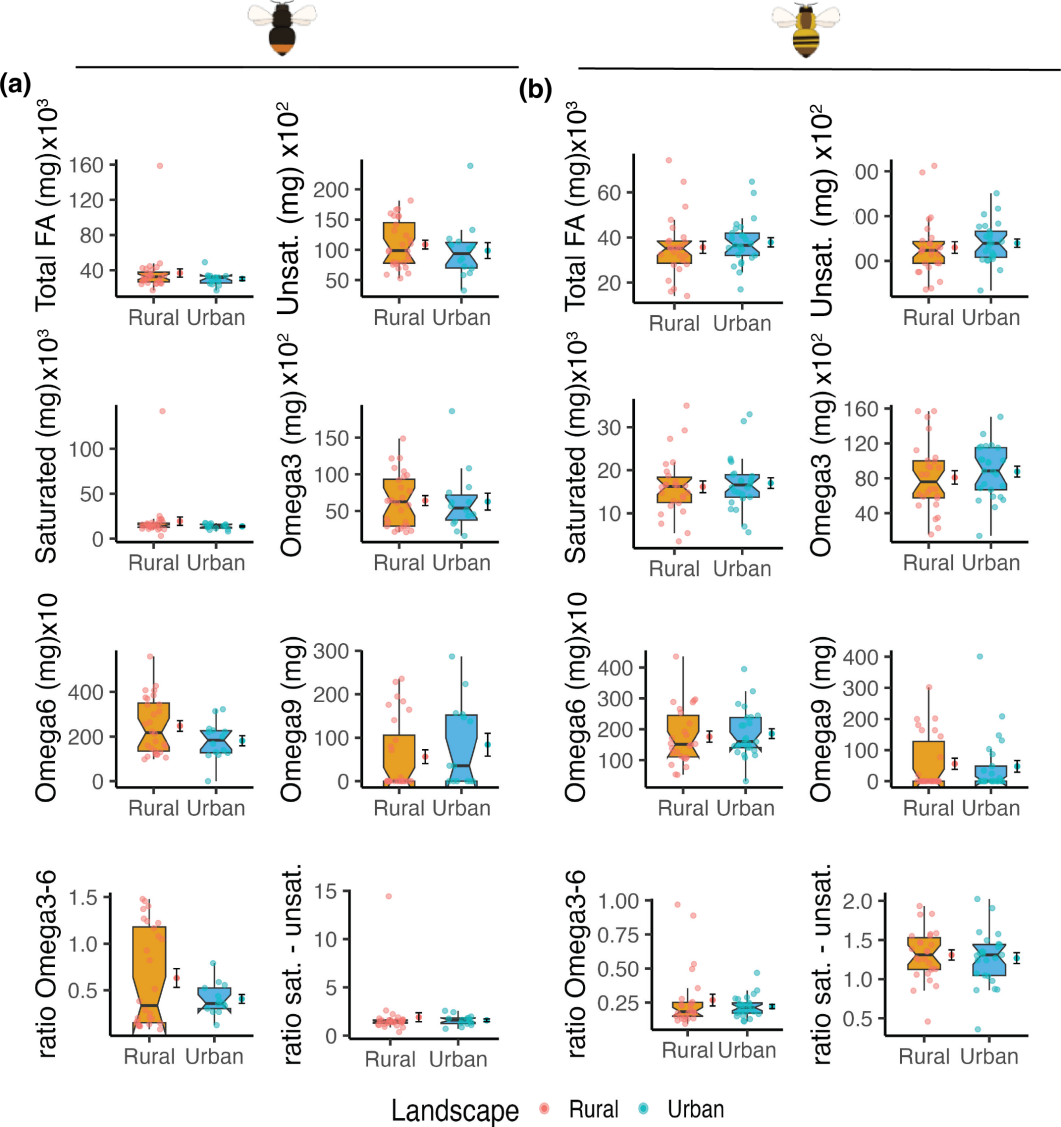
Urban and rural bumblebees have similar fatty acid intake. Boxplots of the fatty acid (FA) composition in the pollen from the leg baskets of urban and rural bumblebees of (a) *Bombus lapidarius* and (b) *Bombus pascuorum*. For simplicity, individual FA have been grouped in eight main groups. Notches indicate the 95% CI of the median. Additionally, on the right side of each boxplot, the mean ± the standard error is also presented. Differences between the means were tested using generalized linear mixed effects models with Benjamini–Hochberg correction for multiple testing.

**Figure 5.**
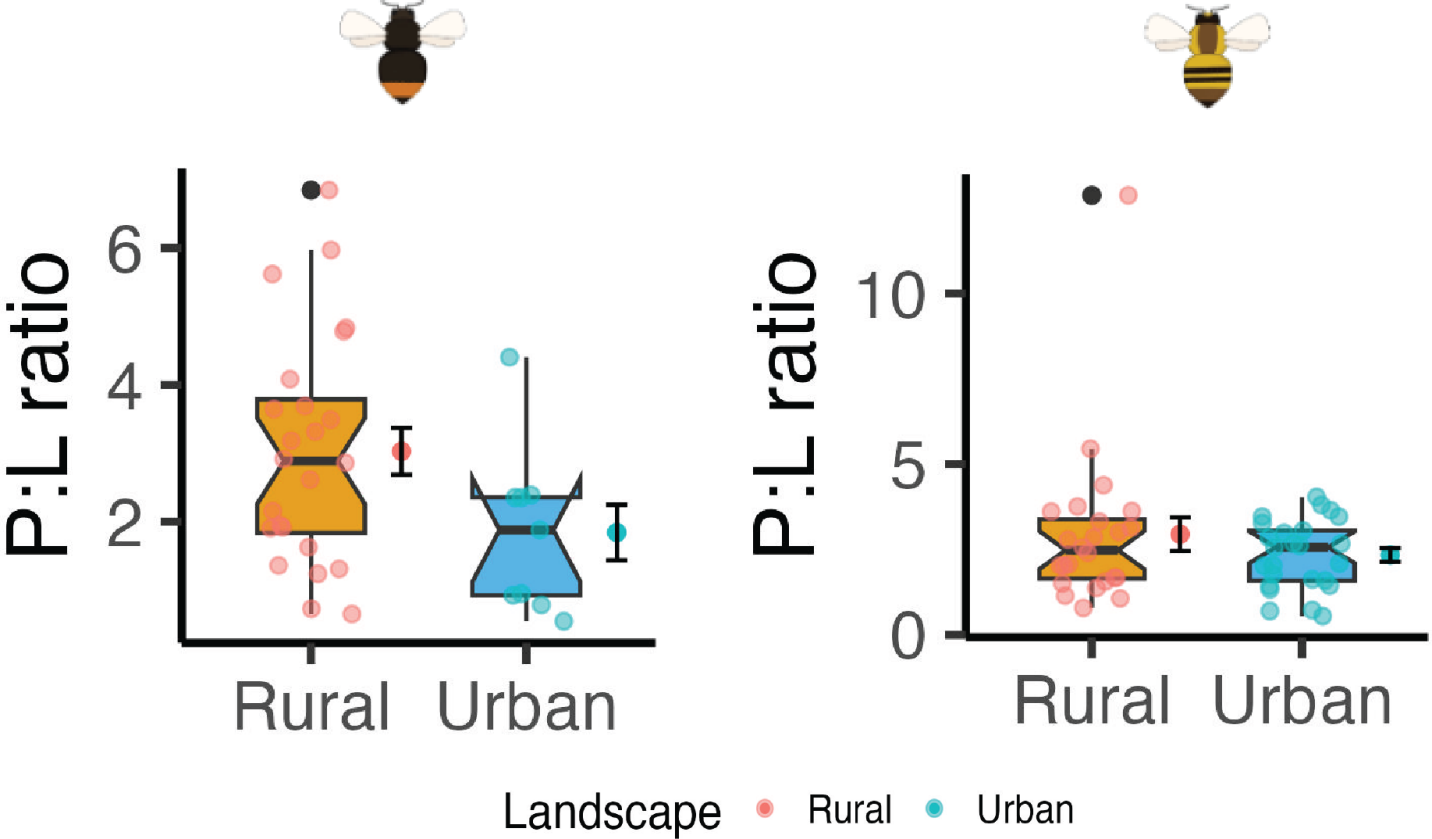
Box plots depicting the differences between the P:L ratio, that is, the ratio of amino acids (P) and fatty acids (L) in urban and rural areas for *Bombus lapidarius* (left) and *B. pascuorum* (right). The mean ± standard deviation is also provided. Notches indicate the 95% CI of the median.

We found a significant decrease in the concentrations of total AA, total essential AA, and total concentrations of non-essential AA in urban compared to rural populations particularly for *B. lapidarius* (figure 3; electronic supplementary material, table S11). Specifically, we observed a 32% decrease in the total AA content, a 32% decrease of essential AA, and a 27% decrease in non-essential AA (figure 3). Regarding FAs, there were no clear differences between urban and rural populations (figure 4; electronic supplementary material, table S11). Interestingly, we found more variation in the AA and FA metrics in rural than in urban populations for both bumblebee species. Finally, we did not observe any differences in the ratio of AAs and FAs between urban and rural areas (figure 5; electronic supplementary material, table S11).

### (d) Pollen-transport patterns

**Figure 6.**
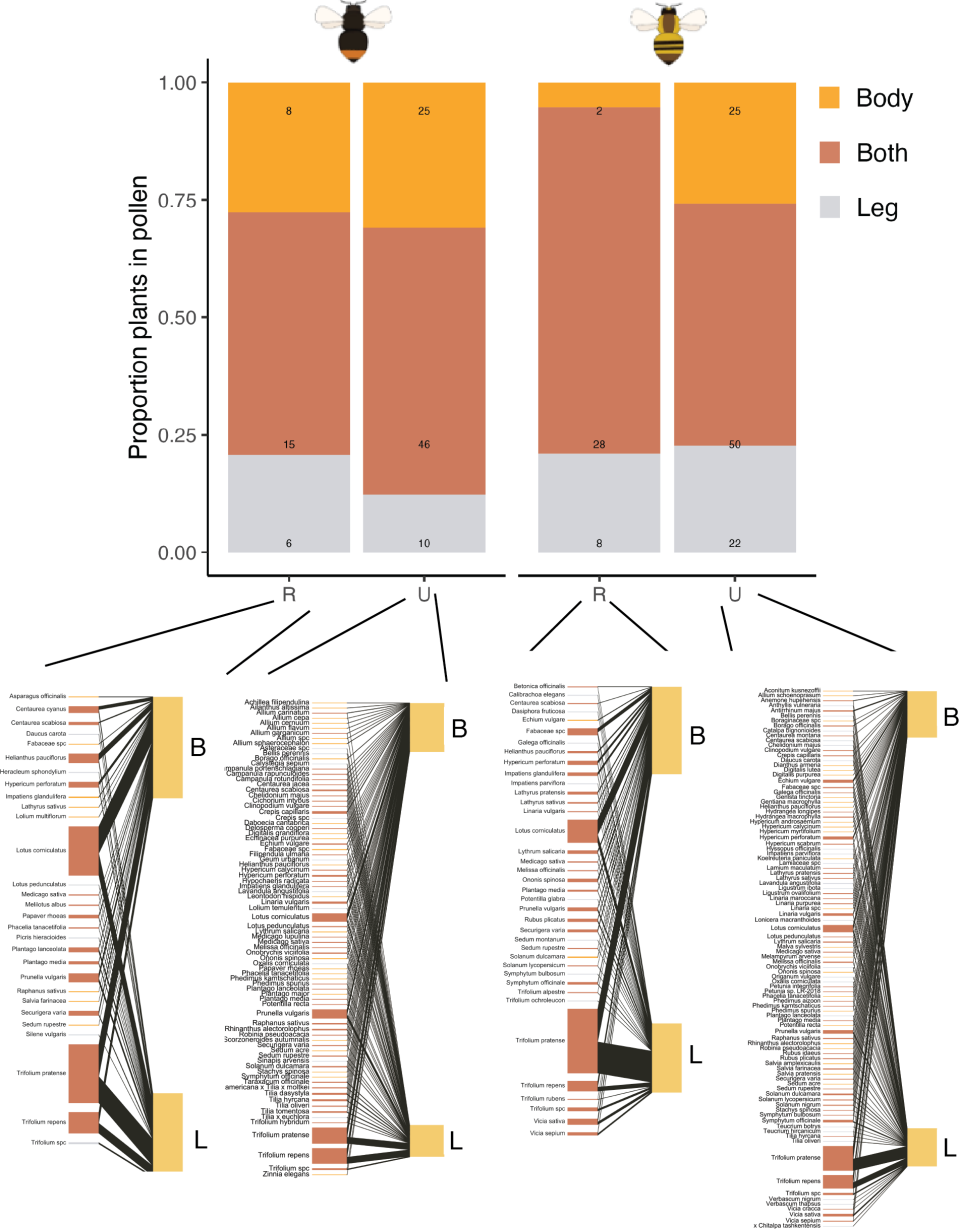
Pollen-transport patterns and networks between pollen-transport structures. B = body; L = leg baskets (corbicula); U = urban landscape; R = rural landscape. The bar plot depicts the proportion of plant species transported only on the body, only on the leg baskets, or in both transportation structures in urban and rural populations of *B. lapidarius* (left) and *B. pascuorum* (right). The number of plant species is also provided. For each bumblebee species and landscape (i.e. urban and rural), the overall bipartite networks (aggregating all individuals) between plant species and bumblebee transportation organs (i.e. body and leg) are also provided. Additional information on pollen-transport can be found in electronic supplementary material, figures S11–S13.

We found contrasting differences in the pollen-transport between bumblebee species, and within species, between urban and rural populations (figure 6; electronic supplementary material, figures S11–S13). For *B. lapidarius*, we found a similar distribution in the proportion of species that were present both in their body and leg basket pollen load (rural: 52%, urban: 57%; figure 6). In addition, the proportion of plant species with pollen transported exclusively on the bumblebee body was also similar between urban and rural populations (rural: 28%, urban: 31%; figure 6). Moreover, in urban populations, the proportion of plant species whose pollen was only transported in leg baskets, and thus, likely less available for pollination, was lower than in rural populations (rural: 20%, urban: 12%; figure 6). On the other hand, for *B. pascuorum*, we found the proportion of plants with pollen transported in both their body and leg to be much larger in rural than in urban populations (rural: 74%, urban: 52%; figure 6), with the proportion of plant species with pollen transported only on the body being much larger in urban individuals (26%) than in rural (5%). Finally, in *B. pascuorum*, the proportion of plant species with pollen only present in the leg baskets was similar between urban and rural populations (rural: 21%, urban: 22%; figure 6). Regarding the pollen-transport networks, we found urban populations to have larger numbers of links per bumblebee individual (*B. pascuorum*_leg_: 33.97%, *B. lapidarius*_leg_: 30.51%; electronic supplementary material, table S12), and thus, higher generality (*B. pascuorum*_leg_: 14.81%, *B. lapidarius*_leg_: 42.44%; electronic supplementary material, table S12). Furthermore, we found reduced niche overlap (*B. pascuorum*_leg_: −40.32%, *B. lapidarius*_leg_: −33.33%; electronic supplementary material, table S12).

## 4. Discussion

Species dietary patterns and pollen-transport patterns are still little investigated, but they are critical to understand how species cope with anthropogenic pressures and interact with resources in urban and rural areas. Overall, we found evidence for our first scenario of diet maintenance: larger floral resources did increase diet breadth in cities but did not translate into better nutrient intake. In that regard, we found two responses linked to our scenarios of urban maintenance and urban disadvantage: (i) bumblebees maintain the acquisition of certain macronutrients such as FAs in both species and AAs for *B. pascuorum*, and (ii) urban populations have reduced content of certain nutrients, specifically, AAs in *B. lapidarius.* Overall, this agrees with previous findings indicating that bumblebees might primarily focus on FA rather than AA intake [15].

Our results support the diet diversification hypothesis for diet breadth [11,12], that is, that more diverse foraging landscapes diversify dietary patterns regarding diet breadth. Urban bumblebees had broader diet breadths than their rural counterparts. This is likely a consequence of two key changes in urban plant communities: (i) an increase in the number of species, and (ii) a more even distribution of plant species within the communities [56]. Enhanced food resources have been documented to expand diet breadth in urban vertebrates for both herbivores and predators [57,58], which might be extensible to generalist insect florivores. While both species show a preference for Fabaceae plants [59], this diet conservatism is stronger in rural populations than in urban ones, indicating that diet preferences can adapt to different environments [60]. Finally, the diet diversification hypothesis was not supported for nutrient intake, with bumblebees apparently maintaining nutrient intake.

Our findings show that bumblebees can secure an adequate nutrient intake across variable landscapes, supporting the nutrient intake maintenance scenario for *B. pascuorum,* and for *B. lapidarius* regarding FAs. Leaving aside the influence of the environment or biotic interactions, our findings support prior work showing that bumblebees are restricted in their nutrient requirements, which forces them to regulate their intake [15] resulting in specific strategies to achieve it [1,61]. There are several documented strategies expected to optimize nutrient intake in bumblebees, regarding their cognition and learning skills (e.g. [62]). Consequently, this might allow generalist bumblebee species to cope with the variations in plant communities such as the ones observed between urban and rural areas. Nonetheless, besides bumblebees’ intrinsic features, there are additional explanations regarding how urban and rural areas might be leading to the nutrient intake maintenance scenario.

Urban and rural areas might have equivalent optimal foraging landscapes and then, diet breadth expansion might emerge from neutral processes. Particularly, this can be expected if (i) the reduction in plant dominance is not affecting preferred plant species (optimal resources), or, if so, if this is compensated by the increase in plant diversity with functionally redundant species, and (ii) if resources remain accessible and sufficiently abundant in the cityscape surrounding bumblebee colonies. If so, the expansion of the diet breadth might even be reinforced by having additional benefits for the stability and resilience of bumblebee colonies (e.g. reducing the dependence on specific plant taxa and diluting toxic pollen [63]).

Alternatively, our results might indicate that urban areas, rural areas or both have suboptimal conditions, enforcing diet diversification. Suboptimal conditions might make key floral resources scarcer, less accessible and/or of lower quality, incurring higher energetic costs (searching and handling resources, competition) with potential consequences on individual and colony fitness [64], as seen also in solitary bees [1]. Suboptimal rural foraging landscapes can emerge from impoverished plant communities due to land-use changes and intensity. Suboptimal urban foraging landscapes might be a consequence of changes in diversity distribution and community structure. Particularly, when preferred optimal resources are reduced and are not compensated by the addition of other plant species. While cities are associated with increasing plant diversity, resulting from multiple habitat types and levels of human facilitation [65], the diversification effect might be counteracted by the addition of plant species of little or no value for bumblebees. In fact, part of the urban plant communities arises from cultivation, including plants that are not necessarily nectar and pollen hosts for pollinators [66]. The generalist diet of bumblebees might enable them to better use such resources than other pollinating insects, but restrictions can still be expected when certain degrees of diet conservatism exist [2]. Notably, we found low contributions of non-native species in both bumblebee species, indicating potential constraints in the use of novel plant resources. Finally, suboptimal foraging landscapes are expected to intensify competition for optimal resources (e.g. among wild pollinators [67], with managed bees [68]), further forcing individuals to switch their diet to suboptimal resources to avoid the negative costs of competition and approximate to optimal foraging. In that regard, our pollen-transport networks indicate more dissimilar interaction networks in urban bumblebees, with individuals interacting with a larger, more variable number of plants. Increasing diet breadth has been identified as a mechanism triggered with higher local abundances of bumblebees, potentially as a mechanism to avoid competition [69,70].

Simultaneously, we found support for the urban disadvantage scenario with regard to AAs in *B. lapidarius*. The chances of obtaining such macronutrients might be lower in urban foraging landscapes. Specifically, given that *B. lapidarius* and *B. pascuorum* have strong preference for Fabaceae and that *B. pascuorum* maintained AA content, the difference in the AA content could reflect more competitive landscapes for *B. lapidarius*. Interestingly, Eggenberger *et al*. [23], who studied morphological trait variability in the same individuals and species, found bimodality in the proboscis length within urban populations in *B. pascuorum* but not for *B. lapidarius*, which could be explained by a better genetic mixing across landscapes in *B. lapidarius* [71]. Bimodality in the tongue length might enable bumblebees to better cope with the conditions of novel foraging landscapes (e.g. resource availability [72]). Nonetheless, it is worth considering that nutrient intake regulation might be more focused on the FAs or on the ratios between lipids and proteins, which might make AAs content by itself a worse indicator of diet quality.

Finally, the results on pollen-transport patterns on *B. pascuorum*, transporting more unique plants in the body in urban individuals than in rural ones, might also reflect foraging challenges in cities. Bumblebees, specially generalists, can learn how to use and access new feeding resources [19,73]. However, bumblebee learning capabilities have some constraints due to the nature of the associative learning between floral cues, rewards and foraging decisions [62,74]. Diversified urban floral types might increase the variability of floral rewards, decreasing the ability to discriminate between suitable and non-suitable plants [62]. Therefore, urban bumblebees might get exposed to flowers of non-targeted plants in the process of learning and maximizing their foraging strategies and the energy gain [75]. For example, we found *B. pascuorum* to have larger proportions of pollen transported only in the body in cities, perhaps suggesting more learning trials and contact with non-targeted plant species.

Our study had some limitations that constrained assessment of the importance of intraspecific trait variability in explaining the variability in the diet breadth between urban and rural bumblebees. Variation in body size and tongue length can be expected to influence foraging of bumblebees as they influence flying distance, amount of pollen that can be carried, and how efficiently flowers can be handled [76]. The lack of findings in our study seems to indicate that intraspecific trait variation may be a consequence of physiological factors rather than to diet ones. Furthermore, the pollen carried by the sampled individuals represents a snapshot of the current foraging trip, and that individual foraging is dynamic in space and time. For example, it is important to consider that bumblebee foragers of the same colony can exhibit different fidelity on their foraging sites at the individual level [77]. Future studies should include data on the structure of floral resources and their availability, abundances of other competing pollinators, bumblebee flight distances in relation to the forage landscape and on bumblebee health and fitness [78] to analyse the contribution of different mechanisms driving dietary patterns in anthropogenic landscapes. In that regard, our study represents a first step towards understanding the factors shaping animal foraging in anthropogenically modified ecosystems. Finding out to what degree species can handle novel ecosystems is a necessary step towards improved conservation, particularly in the context of the Post-2020 Global Biodiversity Framework [79], where some of the efforts are aimed at reducing the adverse effects of human-modified ecosystems on biodiversity and its contributions to people.

## Data Availability

Data and code for this article are available on Dryad [80]. Supplementary material is available online [81].

## References

[1] Peters B, Keller A, Leonhardt SD. 2022 Diets maintained in a changing world: does land‐use intensification alter wild bee communities by selecting for flexible generalists? Ecol. Evol. **12**, e8919. (10.1002/ece3.8919) 35600696 PMC9108308

[2] Wood TJ *et al*. 2021 Global patterns in bumble bee pollen collection show phylogenetic conservation of diet. J. Anim. Ecol. **90**, 2421–2430. (10.1111/1365-2656.13553) 34096055

[3] Tew NE, Baldock KCR, Vaughan IP, Bird S, Memmott J. 2022 Turnover in floral composition explains species diversity and temporal stability in the nectar supply of urban residential gardens. J. Appl. Ecol. **59**, 801–811. (10.1111/1365-2664.14094)

[4] Fretwell SD, Lucas HL. 1969 On territorial behavior and other factors influencing habitat distribution in birds. I. Theoretical development. Acta Biotheor. **19**, 16–36. (10.1007/BF01601953)

[5] Goulson D. 1999 Foraging strategies of insects for gathering nectar and pollen, and implications for plant ecology and evolution. Perspect. Plant Ecol. Evol. Syst. **2**, 185–209. (10.1078/1433-8319-00070)

[6] Pioltelli E, Guzzetti L, Ouled Larbi M, Labra M, Galimberti A, Biella P. 2024 Landscape fragmentation constrains bumblebee nutritional ecology and foraging dynamics. Landsc. Urban Plan. **247**, 105075. (10.1016/j.landurbplan.2024.105075)

[7] Dreisig H. 1995 Ideal free distributions of nectar foraging bumblebees. Oikos **72**, 161. (10.2307/3546218)

[8] Redhead JW, Dreier S, Bourke AFG, Heard MS, Jordan WC, Sumner S, Wang J, Carvell C. 2016 Effects of habitat composition and landscape structure on worker foraging distances of five bumble bee species. Ecol. Appl. **26**, 726–739. (10.1890/15-0546) 27411246

[9] Timberlake TP, Vaughan IP, Memmott J. 2019 Phenology of farmland floral resources reveals seasonal gaps in nectar availability for bumblebees. J. Appl. Ecol. **56**, 1585–1596. (10.1111/1365-2664.13403)

[10] Hülsmann M, von Wehrden H, Klein AM, Leonhardt SD. 2015 Plant diversity and composition compensate for negative effects of urbanization on foraging bumble bees. Apidologie **46**, 760–770. (10.1007/s13592-015-0366-x)

[11] Jha S, Kremen C. 2013 Resource diversity and landscape-level homogeneity drive native bee foraging. Proc. Natl Acad. Sci. USA **110**, 555–558. (10.1073/pnas.1208682110) 23267118 PMC3545746

[12] Kaluza BF, Wallace H, Keller A, Heard TA, Jeffers B, Drescher N, Blüthgen N, Leonhardt SD. 2017 Generalist social bees maximize diversity intake in plant species‐rich and resource‐abundant environments. Ecosphere **8**, e01758. (10.1002/ecs2.1758)

[13] Moerman R, Vanderplanck M, Fournier D, Jacquemart A, Michez D. 2017 Pollen nutrients better explain bumblebee colony development than pollen diversity. Insect Conserv. Divers. **10**, 171–179. (10.1111/icad.12213)

[14] Rivest S, Forrest JRK. 2020 Defence compounds in pollen: why do they occur and how do they affect the ecology and evolution of bees? New Phytol. **225**, 1053–1064. (10.1111/nph.16230) 31569278

[15] Ruedenauer FA, Raubenheimer D, Kessner‐Beierlein D, Grund‐Mueller N, Noack L, Spaethe J, Leonhardt SD. 2020 Best be(e) on low fat: linking nutrient perception, regulation and fitness. Ecol. Lett. **23**, 545–554. (10.1111/ele.13454) 31943632

[16] Vaudo AD, Patch HM, Mortensen DA, Tooker JF, Grozinger CM. 2016 Macronutrient ratios in pollen shape bumble bee (Bombus impatiens) foraging strategies and floral preferences. Proc. Natl Acad. Sci. USA **113**, E4035–E4042. (10.1073/pnas.1606101113) 27357683 PMC4948365

[17] Rosenberger NM, Aizen MA, Dickson RG, Harder LD. 2022 Behavioural responses by a bumble bee to competition with a niche‐constructing congener. J. Anim. Ecol. **91**, 580–592. (10.1111/1365-2656.13646) 34862619 PMC9305565

[18] Sponsler DB, Requier F, Kallnik K, Classen A, Maihoff F, Sieger J, Steffan-Dewenter I. 2022 Contrasting patterns of richness, abundance, and turnover in mountain bumble bees and their floral hosts. Ecology **103**, 1–15. (10.1002/ecy.3712) 35363383

[19] Sponsler D, Kallnik K, Requier F, Classen A, Maihoff AF, Sieger J, Steffan‐Dewenter I. 2022 Floral preferences of mountain bumble bees are constrained by functional traits but flexible through elevation and season. Oikos **2022**, e08902. (10.1111/oik.08902)

[20] Cuff JP, Evans DM, Vaughan IP, Wilder SM, Tercel MPTG, Windsor FM. 2024 Networking nutrients: how nutrition determines the structure of ecological networks. J. Anim. Ecol. **93**, 974–988. (10.1111/1365-2656.14124) 38946110

[21] Ellis EE, Edmondson JL, Maher KH, Hipperson H, Campbell SA. 2023 Negative effects of urbanisation on diurnal and nocturnal pollen‐transport networks. Ecol. Lett. **26**, 1382–1393. (10.1111/ele.14261) 37272470 PMC10946945

[22] Vaudo AD *et al*. 2020 Pollen protein: lipid macronutrient ratios may guide broad patterns of bee species floral preferences. Insects **11**, 132. (10.3390/insects11020132) 32085627 PMC7074338

[23] Eggenberger H, Frey D, Pellissier L, Ghazoul J, Fontana S, Moretti M. 2019 Urban bumblebees are smaller and more phenotypically diverse than their rural counterparts. J. Anim. Ecol. **88**, 1522–1533. (10.1111/1365-2656.13051) 31233621

[24] Biella P, Tommasi N, Guzzetti L, Pioltelli E, Labra M, Galimberti A. 2022 City climate and landscape structure shape pollinators, nectar and transported pollen along a gradient of urbanization. J. Appl. Ecol. **59**, 1586–1595. (10.1111/1365-2664.14168)

[25] Vaudo AD, Dyer LA, Leonard AS. 2024 Pollen nutrition structures bee and plant community interactions. Proc. Natl Acad. Sci. USA **121**, e2317228120. (10.1073/pnas.2317228120) 38190523 PMC10801918

[26] Trinkl M, Kaluza BF, Wallace H, Heard TA, Keller A, Leonhardt SD. 2020 Floral species richness correlates with changes in the nutritional quality of larval diets in a stingless bee. Insects **11**, 125. (10.3390/insects11020125) 32075297 PMC7073955

[27] Casanelles-Abella J, Selva S, Keller A, Ruedenauer F, Fournier B, Leonhardt S, Moretti M. 2025 Data on the diet and nutrition of urban and rural bumblebees. Sci. Data **12**, 1–14. (10.1038/s41597-025-04585-w) 39962107 PMC11832771

[28] Campos MG *et al*. 2021 Standard methods for pollen research. J. Apic. Res. **60**, 1–109. (10.1080/00218839.2021.1948240)

[29] Sickel W, Ankenbrand MJ, Grimmer G, Holzschuh A, Härtel S, Lanzen J, Steffan-Dewenter I, Keller A. 2015 Increased efficiency in identifying mixed pollen samples by meta-barcoding with a dual-indexing approach. BMC Ecol **15**, 1–9. (10.1186/s12898-015-0051-y) 26194794 PMC4509727

[30] Leonhardt SD, Peters B, Keller A. 2022 Do amino and fatty acid profiles of pollen provisions correlate with bacterial microbiomes in the mason bee Osmia bicornis? Phil. Trans. R. Soc. B **377**, 20210171. (10.1098/rstb.2021.0171) 35491605 PMC9058536

[31] Roulston TH, Cane JH. 2000 Pollen nutritional content and digestibility for animals. Plant Syst. Evol. **222**, 187–209. (10.1007/bf00984102)

[32] Kriesell L, Hilpert A, Leonhardt SD. 2017 Different but the same: bumblebee species collect pollen of different plant sources but similar amino acid profiles. Apidologie **48**, 102–116. (10.1007/s13592-016-0454-6)

[33] Villagómez GN, Brachvogel RC, Kárpáti Z, Leonhardt SD, Schmitt T, Ruedenauer FA. 2023 A common protocol for reliable comparison of pollen fatty acid profiles: highlighting pitfalls and proposing a methodology for ecological research. Front. Ecol. Evol. **11**, 1141832. (10.3389/fevo.2023.1141832)

[34] Manning R. 2001 Fatty acids in pollen: a review of their importance for honey bees. Bee World **82**, 60–75. (10.1080/0005772x.2001.11099504)

[35] Arien Y, Dag A, Zarchin S, Masci T, Shafir S. 2015 Omega-3 deficiency impairs honey bee learning. Proc. Natl Acad. Sci. USA **112**, 15761–15766. (10.1073/pnas.1517375112) 26644556 PMC4697434

[36] Nicolson SW. 2011 Bee food: the chemistry and nutritional value of nectar, pollen and mixtures of the two. African Zoology **46**, 197–204. (10.1080/15627020.2011.11407495)

[37] Global Biodiversity Information Facility. 2022 Occurrence download (10.15468/DL.KKWG72)

[38] InfoFlora. 2022 The National Data and Information Center on the Swiss Flora (Swiss National Databank of Vascular Plants). See https://www.infoflora.ch.

[39] Ornai A, Keasar T. 2020 Floral complexity traits as predictors of plant-bee interactions in a Mediterranean pollination web. Plants **9**, 1432. (10.3390/plants9111432) 33114435 PMC7694153

[40] Casanelles-Abella J *et al*. 2021 A dataset of the flowering plants (Angiospermae) in urban green areas in five European cities. Data Brief **37**, 107243. (10.1016/j.dib.2021.107243) 34307807 PMC8258796

[41] Filipiak M, Walczyńska A, Denisow B, Petanidou T, Ziółkowska E. 2022 Phenology and production of pollen, nectar, and sugar in 1612 plant species from various environments. Ecology **103**, 2021–2022. (10.1002/ecy.3705) 35362098

[42] Tew NE, Baldock KCR, Morten JM, Bird S, Vaughan IP, Memmott J. 2023 A dataset of nectar sugar production for flowering plants found in urban green spaces. Ecol. Solutions Evid **4**, e12248. (10.1002/2688-8319.12248)

[43] R Core Team. 2023 R: a language and environment for statistical computing. R Foundation for Statistical Computing. See https://www.R-project.org/.

[44] Kaplan H, Hill K. 2017 The evolutionary ecology of food acquisition. In Evolution, ecology and human behavior (ed. EA Smith), pp. 167–202. New York, NY: Routledge.

[45] Wood TJ, Roberts SPM. 2017 An assessment of historical and contemporary diet breadth in polylectic Andrena bee species. Biol. Conserv **215**. (10.1016/j.biocon.2017.09.009)

[46] Laliberté E, Legendre P. 2010 A distance‐based framework for measuring functional diversity from multiple traits. Ecology **91**, 299–305. (10.1890/08-2244.1) 20380219

[47] Kembel SW, Cowan PD, Helmus MR, Cornwell WK, Morlon H, Ackerly DD, Blomberg SP, Webb CO. 2010 Picante: R tools for integrating phylogenies and ecology. Bioinformatics **26**, 1463–1464. (10.1093/bioinformatics/btq166) 20395285

[48] Jin Y, Qian H. 2019 V.PhyloMaker: an R package that can generate very large phylogenies for vascular plants. Ecography **42**, 1353–1359. (10.1111/ecog.04434) PMC9363651 35967255

[49] Lefcheck JS. 2016 piecewiseSEM: piecewise structural equation modelling in r for ecology, evolution, and systematics. Methods Ecol. Evol **7**. (10.1111/2041-210x.12512)

[50] Shipley B. 2016 Cause and correlation in biology: a user’s guide to path analysis, structural equations and causal inference with R. In Cause correl. biol. Cambridge: Cambridge University Press. (10.1017/CBO9781139979573)

[51] Casanelles‐Abella J, Fontana S, Fournier B, Frey D, Moretti M. 2023 Low resource availability drives feeding niche partitioning between wild bees and honeybees in a European city. Ecol. Appl **33**, 1–17. (10.1002/eap.2727) PMC10077915 36054537

[52] Shipley B. 2013 The AIC model selection method applied to path analytic models compared using a d‐separation test. Ecology **94**, 560–564. (10.1890/12-0976.1) 23687881

[53] Dormann C, Gruber B, Fruend J. 2008 Introducing the bipartite package: analysing ecological networks. R News **8**, 8–11.

[54] Csardi G, Nepusz T. 2006 igraph: network analysis and visualization. See https://cran.r-project.org/web/packages/igraph/.

[55] Pansu J *et al*. 2022 The generality of cryptic dietary niche differences in diverse large-herbivore assemblages. Proc. Natl Acad. Sci. USA **119**. (10.1073/pnas.2204400119) PMC9436339 35994662

[56] Faeth SH, Bang C, Saari S. 2011 Urban biodiversity: patterns and mechanisms. Ann. N. Y. Acad. Sci. **1223**, 69–81. (10.1111/j.1749-6632.2010.05925.x) 21449966

[57] Anders JL, Mychajliw AM, Moustafa MAM, Mohamed WMA, Hayakawa T, Nakao R, Koizumi I. 2022 Dietary niche breadth influences the effects of urbanization on the gut microbiota of sympatric rodents. Ecol. Evol. **12**, e9216. (10.1002/ece3.9216) 36177145 PMC9463044

[58] Gámez-Virués S *et al*. 2015 Landscape simplification filters species traits and drives biotic homogenization. Nat. Commun. **6**, 8568. (10.1038/ncomms9568) 26485325 PMC4634213

[59] Timberlake TP, de Vere N, Jones LE, Vaughan IP, Baude M, Memmott J. 2024 Ten‐a‐day: bumblebee pollen loads reveal high consistency in foraging breadth among species, sites and seasons. Ecol. Sol. and Evidence **5**, e12360. (10.1002/2688-8319.12360)

[60] Ruedenauer FA, Spaethe J, Leonhardt SD. 2016 Hungry for quality—individual bumblebees forage flexibly to collect high-quality pollen. Behav. Ecol. Sociobiol. **70**, 1209–1217. (10.1007/s00265-016-2129-8)

[61] Ruedenauer FA, Spaethe J, Leonhardt SD. 2015 How to know which food is good for you: bumblebees use taste to discriminate between different concentrations of food differing in nutrient content. J. Exp. Biol. **218**, 2233–2240. (10.1242/jeb.118554) 26202778

[62] Hemingway CT, Leonard AS, MacNeill FT, Pimplikar S, Muth F. 2024 Pollinator cognition and the function of complex rewards. Trends Ecol. Evol. **39**, 1047–1058. (10.1016/j.tree.2024.06.008) 39019730

[63] Eckhardt M, Haider M, Dorn S, Müller A. 2014 Pollen mixing in pollen generalist solitary bees: a possible strategy to complement or mitigate unfavourable pollen properties? J. Anim. Ecol. **83**, 588–597. (10.1111/1365-2656.12168) 24164651

[64] Theodorou P, Kühn O, Baltz LM, Wild C, Rasti SL, Bucksch CR, Strohm E, Paxton RJ, Kurze C. 2022 Bumble bee colony health and performance vary widely across the urban ecosystem. J. Anim. Ecol. **91**, 2135–2148. (10.1111/1365-2656.13797) 36002939

[65] Swan CM, Pickett STA, Szlavecz K, Warren P, Willey KT. 2011 Biodiversity and community composition in urban ecosystems: coupled human, spatial, and metacommunity processes. In Urban ecology (eds JH Breuste, T Elmqvist, G Guntenspergen, P James, NE McIntyre), pp. 179–186. Oxford, UK: Oxford University Press. (10.1093/acprof:oso/9780199563562.003.0021)

[66] Garbuzov M, Ratnieks FLW. 2014 Quantifying variation among garden plants in attractiveness to bees and other flower‐visiting insects. Funct. Ecol. **28**, 364–374. (10.1111/1365-2435.12178)

[67] Sponsler D, Iverson A, Steffan‐Dewenter I. 2023 Pollinator competition and the structure of floral resources. Ecography **2023**, e06651. (10.1111/ecog.06651)

[68] Wignall VR, Campbell Harry I, Davies NL, Kenny SD, McMinn JK, Ratnieks FLW. 2020 Seasonal variation in exploitative competition between honeybees and bumblebees. Oecologia **192**, 351–361. (10.1007/s00442-019-04576-w) 31840190 PMC7002462

[69] Fontaine C, Collin CL, Dajoz I. 2008 Generalist foraging of pollinators: diet expansion at high density. J. Ecol. **96**, 1002–1010. (10.1111/j.1365-2745.2008.01405.x)

[70] Glenny WR, Runyon JB, Burkle LA. 2024 Bumble bee diet breadth increases with local abundance and phenophase duration, not intraspecific variation in body size. Oecologia **205**, 149–162. (10.1007/s00442-024-05560-9) 38796612 PMC11144151

[71] Theodorou P, Radzevičiūtė R, Kahnt B, Soro A, Grosse I, Paxton RJ. 2018 Genome-wide single nucleotide polymorphism scan suggests adaptation to urbanization in an important pollinator, the red-tailed bumblebee (Bombus lapidarius L.). Proc. R. Soc. B **285**, 20172806. (10.1098/rspb.2017.2806) PMC5936727 29669900

[72] Owen RE, Harder LD. 1995 Heritable allometric variation in bumble bees: opportunities for colony‐level selection of foraging ability. J. Evol. Biol. **8**, 725–738. (10.1046/j.1420-9101.1995.8060725.x)

[73] Zhou Y, Ding S, Liao C, Wu J, Chittka L, Solvi C, Peng F. 2024 Bumble bees’ food preferences are jointly shaped by rapid evaluation of nectar sugar concentration and viscosity. Anim. Behav. **210**, 419–427. (10.1016/j.anbehav.2024.02.006)

[74] Rands SA, Whitney HM, Hempel de Ibarra N. 2023 Multimodal floral recognition by bumblebees. Curr. Opin. Insect Sci. **59**, 101086. (10.1016/j.cois.2023.101086) 37468044

[75] Pattrick JG, Symington HA, Federle W, Glover BJ. 2023 Bumblebees negotiate a trade-off between nectar quality and floral biomechanics. iScience **26**, 108071. (10.1016/j.isci.2023.108071) 38107877 PMC10725025

[76] Chole H, Woodard SH, Bloch G. 2019 Body size variation in bees: regulation, mechanisms, and relationship to social organization. Curr. Opin. Insect Sci. **35**, 77–87. (10.1016/j.cois.2019.07.006) 31426016

[77] Heinrich B. 1976 The foraging specializations of individual bumblebees. Ecol. Monogr. **46**, 105–128. (10.2307/1942246)

[78] Parreño MA *et al*. 2022 Critical links between biodiversity and health in wild bee conservation. Trends Ecol. Evol. **37**, 309–321. (10.1016/j.tree.2021.11.013) 34955328

[79] Open-Ended Working Group on the Post-2020 Global Biodiversity Framework. 2022 Post-2020 Global Biodiversity Framework. See https://www.cbd.int/doc/c/409e/19ae/369752b245f05e88f760aeb3/wg2020-05-l-02-en.pdf.

[80] Selva S. 2025 Data for: Urban bumblebees diversify their foraging strategy to maintain nutrient intake. Dryad Digital Repository. (10.5061/dryad.59zw3r2jn) PMC12092122 40393485

[81] Selva S, Moretti M, Ruedenauer FA, Keller A, Fournier B, Leonhardt SD *et al*. 2025 Supplementary material from: Urban bumblebees diversify their foraging strategy to maintain nutrient intake. Figshare. (10.6084/m9.figshare.c.7822964) PMC12092122 40393485

